# Citrate for continuous renal replacement therapy: safer, better and cheaper

**DOI:** 10.1186/s13054-014-0661-3

**Published:** 2014-12-03

**Authors:** Heleen M Oudemans-van Straaten

**Affiliations:** Department of Intensive Care, VU University Medical Center, De Boelelaan 1117, 1081 HZ Amsterdam, The Netherlands

## Abstract

In a previous issue of *Critical Care*, Schilder and colleagues report the results of their multicenter trial (Citrate Anticoagulation Versus Systemic Heparinization; CASH) comparing regional anticoagulation with citrate to heparin anticoagulation. They found that citrate was safer, more efficacious and cheaper than heparin. In contrast to the largest previous trial, however, a survival benefit was not found, which was the primary endpoint of the CASH trial. Different explanations are possible, including selection bias and a lower severity of disease. Selection bias was high: only 6% of the renal replacement therapy patients were included (versus 56% in the previous trial) and exclusion was 56% for increased risk of bleeding, 2.5 times as frequent as in the previous trial. Thus, the trial with survival benefit apparently included more patients with risk of bleeding and also more severely ill patients and these are the groups that potentially benefit the most from citrate. Nevertheless, the CASH trial is the third large randomized trial showing superiority of citrate over heparin, supporting the recommendation of citrate as first choice anticoagulant.

Continuous renal replacement therapy (CRRT) is used for critically ill patients with acute kidney injury in the setting of multiple organ failure. To prevent clotting in the extracorporeal circuit, anticoagulation is required. The commonly used strategies are heparin, causing systemic anticoagulation, and citrate, providing regional anticoagulation of the circuit. As a result, citrate does not increase the patient’s risk of bleeding. On account of this, citrate should be the first choice in critically ill patients. However, many doctors doubt its safety. The time has come to drop this delusion. The recently published multicenter CASH trial (Citrate Anticoagulation versus Systemic Heparinisation) is the third large randomized controlled trial in a row showing superiority of citrate over heparin [1-3]. Citrate was safer, more efficacious and cheaper. In contrast to the OLVG (Onze Lieve Vrouwe Gasthuis) trial [2], however, a survival benefit, which was the primary endpoint of the CASH trial, was not found.

## Differences between the studies

Differences between the trials involve design, selection bias, patient characteristics, type of heparin, modality of CRRT and effect on mortality (Table 1). Remarkably, enrollment in the CASH trial was extremely slow and the study was prematurely discontinued. Despite its multicenter design, it took 6 years to include 139 patients. Only 139 of 2,300 patients with indication for CRRT were included. This 6% enrollment rate profoundly contrasts with the 56% enrollment rate in the OLVG trial. Thus, the selection of patients in the CASH trial was extreme, downgrading its generalizability. The authors suggest that the ‘availability of citrate with its longer filter survival’ raised the threshold for enrollment. However, need for therapeutic anticoagulation and risk of bleeding were the main reasons for exclusion mentioned in the CONSORT diagram. Exclusion rates for need for therapeutic anticoagulation were 19% (432/1,297) in the CASH trial and 7% (26/385) in the OLVG trial (*P* <0.0001). This difference can partially be explained by a different anticoagulation policy. At the time of the OLVG study, atrial fibrillation was not a strict indication for anticoagulation. Exclusion rates for risk of bleeding were 1,297/2,300 (56%) in the CASH trial and 85/385 (22%) in the OLVG trial (*P* <0.0001). Altogether, the CASH trial population differed from the OLVG population, likely including patients with a higher bleeding risk.

**Table 1:** **Comparison between three large randomized controlled trials comparing citrate to heparin anticoagulation for continuous venovenous hemofiltration**

	**CASH trial 2014** [1] **(multicenter)**			**OLVG trial 2009** [2] **(single center)**			**Hetzel trial 2011** [3] **(multicenter)**		
**Excluded (percentage of patients needing CRRT)**	1,297/2,300 (94%)			170/385 (44%)			Not reported		
**Modality**	Predilution CVVH			Postdilution CVVH			Predilution CVVH		
**Groups**	**Citrate**	**Heparin**	***P*** **value**	**Citrate**	**LMWH**	***P*** **value**	**Citrate**	**Heparin**	***P*** **value**
**Number of included patients**	66	73		97	103		87	83	
**Patient characteristics**									
**Age**	67 (36–87)	67 (23–85)		73 (64–79)	73 (67–79)		62 (SD 15)	65 (SD 12)	
**APACHE II**	23 (11–53)	25 (6–43)		28 (27–30)	28 (27–29)		22 (SD 5.1)	22 (SD 5.5)	
**SOFA**	10 (2–19)	11 (3–18)		11 (10–13)	11 (10–14)		10 (SD 3.0)	10 (SD 2.6)	
**Cause of acute kidney injury** ^**a**^									
**Septic**	41%	37%		43%	49%		77%	75%	
**Ischemic (cardiogenic + hypovolemic)**	50%	51%		80%	61%		Not reported		
**Safety**									
**Adverse events needing discontinuation**	5 (8%)	24 (33%)	<0.001	2 (2%)	19 (19%)	<0.001			
**Bleeding percentage** ^**b**^	3 (5%)	10 (14%)	0.09	0 (0%)	16 (16%)	<0.001	5 (5.7%)	12 (14.7%)	0.09
**Citrate accumulation**	4 (6%)			1 (1%)			1 (1%)		
**Efficacy**									
**Circuit survival (hours)** ^**c**^	46 (2–138)	32 (1–72)	0.02	27 (13–47)	26 (15–43)	0.68	38 (SD 23)	26 (SD 19)	<0.001
**Mortality**									
**90-day**	42%	42%	1.00	48%	63%	0.03			
**28-day**	33%	35%	1.00				47%	41%	

Values are median (25th to 75th percentile), means (standard deviation (SD)), number (%). ^a^More causes possible. ^b^Criteria for bleeding differed between studies. ^c^Calculated for the first filter in the CASH trial, and for all filters in the other two. APACHE, Acute Physiology and Chronic Health Evaluation; CASH, Citrate Anticoagulation Versus Systemic Heparinization; CRRT, continuous renal replacement therapy; CVVH, continuous venovenous hemofiltration; LMWH, low molecular weight heparin; OVLG, Onze Lieve Vrouwe Gasthuis; SOFA, Sequential Organ Failure Assessment.

In addition, patients in the OLVG trial were older and more severely ill than in both other trials, explaining the higher overall mortality in the OLVG study (entirely on account of the heparin group), because age and Acute Physiology and Chronic Health Evaluation (APACHE) score were independent predictors of mortality in both studies. Some patients with very low APACHE scores were included in the CASH trial (Table 1). Finally, the CASH protocol used predilution CRRT and supplemented less magnesium.

## Interpretation

We can only speculate whether these differences can explain why citrate did not confer a survival benefit in the CASH trial [1] and Hetzel trial [3] but did in the OLVG trial [2]. In the latter, citrate was especially beneficial in younger patients and those with more severe organ failure, in surgical patients and those with sepsis. Subgroup analysis in the CASH trial did not show significant differences, but some trends were similar: the survival benefit for citrate tended to be higher in younger patients (odds ratio (OR) 0.61, 95% confidence interval (CI) 0.31 to 1.83) and those with higher APACHE score (OR 0.53, 95% CI 0.19 to 1.48). If more patients had been included and the effects were similar, the width of the CI would have been smaller. No survival benefit was seen for citrate in the septic population in the CASH trial, possibly because the more severely ill septic patients were not included in the CASH trial due to thrombocytopenia. These patients likely benefit most from citrate.

## Conclusion

The CASH trial confirms the superiority of citrate in patients without an increased risk of bleeding in terms of safety and efficacy, while the intervention is less costly. Citrate confers an even greater benefit when the risk of bleeding is increased, because CRRT without anticoagulation is really problematic. Randomized studies in this population will, however, never be available. Thus, stubborn objectors: surrender! Citrate is the first choice.

## Abbreviations

APACHE: Acute Physiology and Chronic Health Evaluation
CASH: Citrate Anticoagulation Versus Systemic Heparinization
CI: Confidence interval
CRRT: Continuous renal replacement therapy
OLVG: Onze Lieve Vrouwe Gasthuis
OR: Odds ratio
